# Current Therapies Focused on High-Density Lipoproteins Associated with Cardiovascular Disease

**DOI:** 10.3390/molecules23112730

**Published:** 2018-10-23

**Authors:** Diego Estrada-Luna, María Araceli Ortiz-Rodriguez, Lizett Medina-Briseño, Elizabeth Carreón-Torres, Jeannett Alejandra Izquierdo-Vega, Ashutosh Sharma, Juan Carlos Cancino-Díaz, Oscar Pérez-Méndez, Helen Belefant-Miller, Gabriel Betanzos-Cabrera

**Affiliations:** 1 Instituto Nacional de Cardiología “Ignacio Chávez” Juan Badiano No. 1, Belisario Domínguez Sección 16, 14080 Tlalpan, Mexico City, Mexico; diego.estrada.luna@gmail.com (D.E.-L.); qfbelizabethcm@yahoo.es (E.C.-T.); opmendez@yahoo.com (O.P.-M.); 2 Facultad de Nutrición, Universidad Autónoma del Estado de Morelos, UAEM, Calle Río Iztaccihuatl S/N, Vista Hermosa, 62350 Cuernavaca, Morelos, Mexico; araceli.ortiz@gmail.com; 3 Universidad de la Sierra Sur, UNSIS, Miahuatlán de Porfirio Díaz, 70800 Oaxaca, Mexico; lizmedinab@gmail.com; 4 Área Académica de Medicina, Instituto de Ciencias de la Salud, Universidad Autónoma del Estado de Hidalgo, Carretera Actopan-Tilcuautla, Ex-Hacienda La Concepción S/N, San Agustín Tlaxiaca, 42160 Hidalgo, Mexico; jizquierdovega@gmail.com; 5 Tecnologico de Monterrey, School of Engineering and Sciences, Campus Queretaro, Epigmenio Gonzalez 500, 76130 Queretaro, Mexico; asharma@itesm.mx; 6 Departamento de Microbiología, Escuela Nacional de Ciencias Biológicas del Instituto Politécnico Nacional, 11340 Ciudad de México, Mexico; jccancinodiaz@hotmail.com; 7 Dale Bumpers National Rice Research Center, Stuttgart, AR 72160, USA; drhelenmiller@gmail.com

**Keywords:** lipoproteins, HDL-C, paraoxonase, polyphenols

## Abstract

High-density lipoproteins (HDL) comprise a heterogeneous family of lipoprotein particles divided into subclasses that are determined by density, size and surface charge as well as protein composition. Epidemiological studies have suggested an inverse correlation between High-density lipoprotein-cholesterol (HDL-C) levels and the risk of cardiovascular diseases and atherosclerosis. HDLs promote reverse cholesterol transport (RCT) and have several atheroprotective functions such as anti-inflammation, anti-thrombosis, and anti-oxidation. HDLs are considered to be atheroprotective because they are associated in serum with paraoxonases (PONs) which protect HDL from oxidation. Polyphenol consumption reduces the risk of chronic diseases in humans. Polyphenols increase the binding of HDL to PON1, increasing the catalytic activity of PON1. This review summarizes the evidence currently available regarding pharmacological and alternative treatments aimed at improving the functionality of HDL-C. Information on the effectiveness of the treatments has contributed to the understanding of the molecular mechanisms that regulate plasma levels of HDL-C, thereby promoting the development of more effective treatment of cardiovascular diseases. For that purpose, Scopus and Medline databases were searched to identify the publications investigating the impact of current therapies focused on high-density lipoproteins.

## 1. Lipoproteins: Classes and Functions

Lipoproteins are macromolecular complexes of cellular origin with a structure made up of two parts: the core and the surface. In the core, non-polar lipids are found, such as cholesterol esters, triglycerides, and small amounts of fat-soluble vitamins, which make the core highly hydrophobic. The surface of lipoproteins is composed of amphipathic lipids such as free cholesterol and phospholipids (mainly sphingomyelin and phosphatidylcholine) exhibiting their polar groups to the aqueous medium while their hydrophobic ends are oriented towards the core. This monolayer of lipoproteins is stabilized by a set of proteins called apolipoproteins (Apo) scattered throughout the lipoproteins [1,2,3]. Apolipoproteins, in addition to providing stability to lipoproteins, are enzyme cofactors and contribute to the regulation of the content of intracellular cholesterol [4]. 

**Figure 1:**
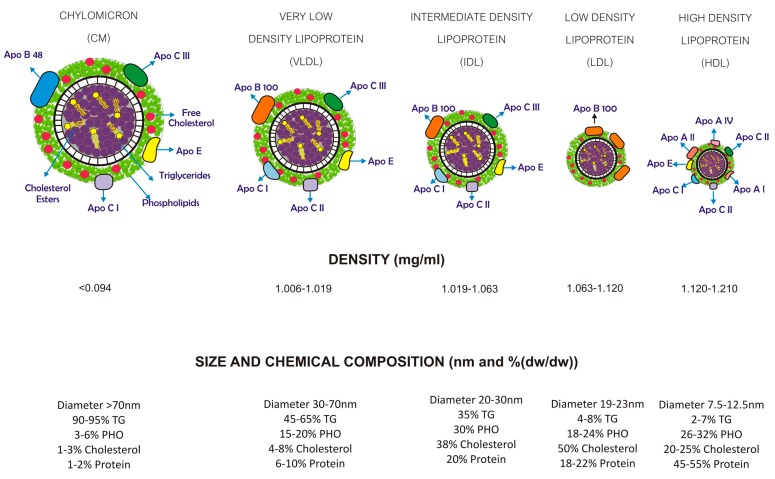
Classes and composition of lipoproteins. The classification criterion is based on the flotation density of the lipoproteins, the proportion of the different lipids and apolipoproteins, and the diameter of the particle. Chylomicrons (CM) are large, triglyceride-rich, and made in the enterocytes. The removal of triglycerides from very low-density lipoproteins (VLDL) results in the formation of intermediate-density lipoprotein (IDL) particles which are enriched in cholesterol and triglycerides and are pro-atherogenic. Low-density lipoproteins (LDL) are enriched only in cholesterol and are the most pro-atherogenic particles. High-density lipoproteins (HDL) are smaller than the other lipoproteins and enriched mainly in proteins. TG = triglycerides; PHO = phospholipids; Apo = apolipoprotein.

The most common way to classify lipoproteins is by density. Based on this, lipoproteins are classified into five classes (lower to higher density; Figure 1): chylomicrons (CM), very low-density lipoproteins (VLDL), intermediate-density lipoproteins (IDL), low-density lipoproteins (LDL), and high-density lipoproteins (HDL) [2,3]. Each of these classes differ in their apolipoprotein contents and their chemical compositions. Lipoproteins are also classified by the physicochemical properties as exhibited by their electrophoretic mobility (high to low) into three classes: α (HDL), pre-β (VLDL) and β (LDL) [5]. Lipoproteins are also grouped by apolipoprotein content: Apo-B100 is found in VLDL, IDL, and LDL; Apo-B48 is found in CM, and Apo-A1 is found in HDL.

Lipoproteins are important in the transport of triglycerides, phospholipids and cholesterol in the body. The study of lipoproteins and their role in the development of various metabolic diseases has increased recently and has resulted in the development of new treatments to regulate the concentration of lipids. The focus has been particularly on the regulation of low-density plasma lipoprotein-cholesterol (LDL-C) levels. Low LDL-C levels can reduce the risk of cardiovascular disease to 25–35% [6,7,8]. This residual risk (65–75%) can in part be explained by lipid abnormalities termed “the atherogenic dyslipidemia complex” which include a reduction in HDL particle numbers and indicating that coronary events can still occur despite low LDL [9,10,11].

HDL is a heterogeneous group of the smallest lipoproteins and the densest (Figure 1) due to their high protein content [3,12]. HDL has been characterized by their density and size by employing laboratory methods such as ultracentrifugation, nuclear magnetic resonance, one-dimensional gel electrophoresis, high-performance liquid chromatography, and ionic mobility [13,14].

The five subfractions of HDL are HDL2a, HDL2b, HDL3a, HDL3b, and HDL3c, according to their physicochemical composition, which indicates their atheroprotective role. Subjects with metabolic syndrome and early cardiovascular heart diseases (CHD) show a low cholesterol efflux by HDL2 and HDL3 within low levels in plasma of these subclasses [15,16]. In subjects with diabetes, HDL2 may be associated with a decrease of carotid artery intima-media thickness [17]. HDL3 is the most abundant form of HDL (approximately 75% of total HDL-C levels), plays an important role in reverse cholesterol transport (RCT), and is considered to be the primary molecule responsible for HDL functions [18]. HDL3 is largely responsible for the inverse association with CHD risk in primary prevention subjects [19]. Much more work is left to be done on the atheroprotective mechanism of HDL subfractions and their relationship to CHD.

However, to complement the study of HDL, second-generation methods have been developed which encapsulate iron beads linked to dextran sulfate coupled to a magnet to facilitate the separation of Apo-B from the HDL fraction. Also, various HPLC methods have been developed to fractionate lipoproteins and their subclasses by size and quantify the cholesterol with enzymatic reagent detection [20,21]. Most recently, another innovative method is nuclear magnetic resonance (NMR); this method has also been effective in quantifying lipoproteins. Jiménez et al. (2018) reported an extensive 600 MHz NMR trial of quantitative lipoprotein measurements in human blood serum and plasma. The variance in measurment internal quality controls was lower than the National Cholesterol Education Program (NCEP) criteria for lipid testing (triglycerides < 2.7%; cholesterol < 2.8%; LDL-cholesterol < 2.8%; HDL-cholesterol < 2.3%), showing exceptional reproducibility for direct quantitation of lipoproteins [22]. This method provides further evidence of the suitability of NMR for high-throughput lipoprotein subcomponent analysis and small molecule quantitation with the excellent reproducibility required for clinical and other regulatory settings [21,22].

### 1.1. HDL Physiological Importance and Metabolism

**Figure 2:**
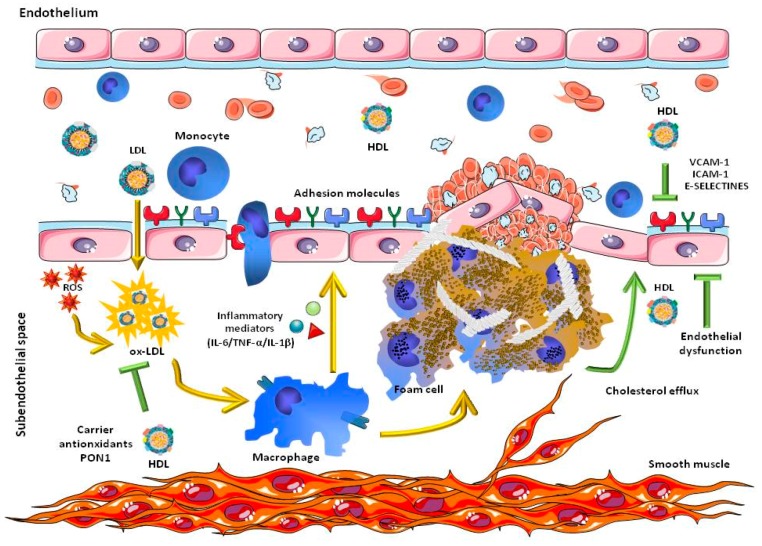
Cardioprotective function of HDL. The main cardioprotective properties of HDL include the inhibition of the oxidation of LDL (ox-LDL) through the activity of paraoxonase 1 (PON1) and the transport of antioxidant molecules, the inhibition of the expression of adhesion molecules in inflammatory processes and the efflux of cholesterol, decreasing the endothelial dysfunction. IL = interleukin; TNF = tumor necrosis factor; ROS = reactive oxygen species. VCAM-1 = vascular cell adhesion molecule 1; ICAM-1 intercellular adhesion molecule 1.

High levels of total cholesterol and LDL-C, along with low levels of HDL-C, are risk factors for the development of cardiovascular diseases, particularly acute myocardial infarction, acute coronary syndrome, and atherosclerosis [18,23,24,25]. In contrast, normal or high HDL levels appear to have anti-atherosclerotic, anti-inflammatory, antioxidant and anti-thrombotic properties, even in the presence of high LDL-C [26,27,28,29] (Figure 2).

The classical explanation for the anti-atherogenic role of HDL is its intravascular metabolism, known as reverse cholesterol transport (RTC). This mechanism consists of exporting the cholesterol from peripheral tissues (macrophage foam cells) and subsequent transport toward the liver for excretion as bile and feces. The synthesis of HDL begins in the liver and small intestine and depends on diverse physicochemical components, mainly apolipoprotein A1 (Apo-Al), which is a major protein component of HDL. Studies have described that the role of HDL in eliminating excess phospholipids and intracellular cholesterol from tissues has been attributed to the Apo-A1 content [30]. The first precursors produced and released from HDL are pre-β1-HDL particles. The enzyme lecithin-cholesterol acyltransferase (LCAT), which is synthesized in the liver, promotes maturation from pre-β HDL to α-HDL, catalyzing the formation of an ester bond between a fatty acid from lecithin (phosphatidylcholine) and a molecule of free cholesterol. Cholesterol esters are more hydrophobic than free cholesterol, so their incorporation into the core of HDL is promoted, pushing more free cholesterol to the surface of HDL [31,32,33,34].

**Figure 3:**
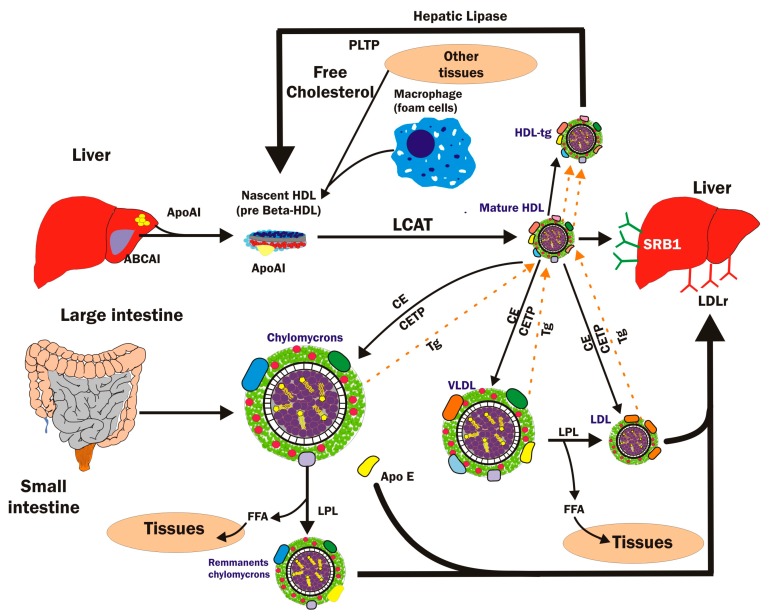
Biogenesis and catabolism (reverse cholesterol transport) of high-density lipoproteins. ABCA1 = ATP binding cassette subfamily A member 1; ApoAl = apolipoprotein Al; ApoE = apolipoprotein E; CE = cholesterol ester; CETP = cholesteryl ester transfer protein; FFA = free fatty acids; LCAT = lecithin-cholesterol acyltransferase; LDL = low density lipoprotein; LDLr = LDL receptor LPL = lipoprotein lipase; PLTP = phospholipid transfer protein; SR-B1 = scavenger receptor class B member 1; Tg = triglycerides; VLDL = very low-density lipoprotein.

The cholesterol ester transporter protein (CETP) exchanges the cholesterol esters in the HDL core with triglycerides present in CM, VLDL, and LDL [32,35]. The resulting increase in the cholesterol ester content of HDL cores induces the formation of the typical spherical shape of these lipoproteins [30,34,36,37] (Figure 3).

Meta-analysis shows that high levels of HDL are associated with a lower risk of cardiovascular disease and pooled relative risk [38,39]. In another meta-analysis of eight statins, trials showed a decreased risk of major cardiovascular events (5387 trial participants; adjusted hazard ratio (HR) of 0.83; 95% confidence interval (CI) of 0.81–0.86 [40]). However, other meta-analyses showed that the increase in HDL-C or HDL-P (HDL particles) via pharmacological manipulation in secondary prevention is not beneficial in randomized clinical trials including 26,858 participants with a follow-up period of 1–6.2 years [41]. Subjects with metabolic syndrome and early cardiovascular heart diseases (CHD) show low cholesterol efflux by HDL2 and HDL3 within low levels in plasma of these subclasses [15,16]. In subjects with diabetes, HDL2 may decrease carotid artery intima-media thickness [17]. HDL3 is the most abundant form of HDL in people with these pathologies (approximately 75% of total HDL-C levels), which plays an important role in reverse cholesterol transport (RCT) and is considered to be the principal subclass of particles responsible for HDL functions [18]. HDL3 is largely responsible for the inverse association with CHD risk in primary prevention subjects [19].

### 1.2. Association of Inflammation and HDL in the Development of Cardiovascular Disease

Studies on the participation of lipoproteins in the development of cardiovascular diseases have uncovered an association with inflammation. Studies evaluating racial and ethnic groups confirm that low HDL-C is an important independent risk factor for cardiovascular events [42,43]. The American Heart Association and the American College of Cardiology consider that the concentration of HDL-C is an important enough predictor of risk of cardiovascular events that it should be evaluated in clinical practice. People who are overweight or obese usually have a decrease in HDL-C and an increase in LDL-C plasma concentrations, both of which are correlated with the risk of cardiovascular disease in patients with rheumatoid arthritis [44] as well as increased inflammatory biomarkers in children with idiopathic arthritis [45,46].

The anti-inflammatory effects of HDL may be critical for protection against different diseases such as diabetes, metabolic syndrome and atherosclerosis [47]. In animal models, the administration of HDL has been shown to decrease atherosclerotic lesions [43]. Normal HDL is capable of preventing LDL oxidation and inflammatory responses induced by LDL [48]. Higher levels of HDL (> 50 mg/dL) increased the anti-inflammatory activity in subjects with diabetes, metabolic syndrome or obesity and decreased carotid intima-media thickness [49,50,51]. Treatments that modulate the lipid profile by increasing HDL and decreasing LDL concentrations may reduce the progression of atherosclerosis in humans [52]. HDL may come to be considered essential for therapeutic strategies to inhibit or reverse the damage of atherosclerotic lesions [35,36].

Some studies have reported a decrease in total HDL-C and specific HDL subfractions in subjects with type 2 diabetes and insulin resistance [53], and in subjects with rheumatoid arthritis with a high risk of CHD [54]. It is hypothesized that the change in the composition of the HDL subfamily is related to the loss of atheroprotective properties, including antioxidant activity [28,29], anti-inflammatory effects, and a lower ability to promote cholesterol efflux [14,55]. To corroborate this hypothesis, Stampfer et al. [56] analyzed the HDL-C and protein content in 246 male patients with and without acute myocardial infarction. The results showed a high correlation between the levels of the smaller HDL subclasses (e.g., HDL3 compared to HDL2) and acute myocardial infarction, suggesting that both total HDL and HDL2 levels confer cardiovascular protection. Similarly, Martin et al. [18] also found that cardiovascular diseases are more associated with low HDL3 levels than with HDL and HDL2 levels. Some specific components of HDL have been found to be risk factors for cardiovascular diseases. While low HDL-C concentration is a risk factor, the best biomarker for cardiovascular disease is a low concentration of Apo-A1 [57], which is found exclusively on HDL particles. 

### 1.3. Inflammation and Antioxidants

Oxidative stress is a condition wherein the cellular production of destructive reactive oxygen species (ROS) exceeds the physiological capacity of endogenous and exogenous antioxidants to inactivate them. ROS damage cells by reacting with various cellular components including carbohydrates, proteins, lipids, and DNA [58]. The cellular damage by oxidative stress is associated with the development of various chronic diseases including cardiovascular disease [59,60,61]. A balance between ROS and antioxidants is essential to reduce the risk of these diseases. Different types of antioxidants (endogenous and exogenous) are involved in the regulatory system to maintain appropriate levels of ROS including: (i) endogenous antioxidants such as albumin, bilirubin, glutathione selenoperoxidase, and uric acid; (ii) antioxidant enzymes such as paraoxonase (PON), superoxide dismutase (SOD), catalase (CAT), glutathione peroxidase (GPx), heme oxygenase-1, NAD(P)H and quinone oxidoreductase; and (iii) dietary antioxidants, including vitamins C and E, carotenoids, and various polyphenol compounds [62,63,64,65].

Consumption of polyphenols from plant extracts and fruits increases antioxidant levels in plasma which protect vasculature and improve anti-inflammatory and lipid profiles, blood pressure, HDL-C, and vascular function. Antioxidants such as vitamins C and E have been shown to partially protect LDL from oxidation in vivo [66,67], as well as by the second line of defense HDL, preventing lipid oxidation through several associated antioxidant enzymes like PON and platelet-activating factor acetylhydrolase (PAF-AH). Other HDL-associated protein elements like Apo-AII, Apo-AIV, Apo J, Apo L-1, and Apo F have been reported to give a better inhibition of expression and circulating levels of some adhesion molecules (vascular cell adhesion molecule 1/intercellular adhesion molecule 1, VCAM-1/ICAM-1/E-selectines) [68,69,70,71,72].

### 1.4. Anti-Thrombotic

There is a connection between some inflammatory disease and thrombosis, showing an increase of prothrombotic molecules and platelet activation [73], diverse atherothrombotic diseases such as acute coronary syndrome, stroke, or peripheral arterial disease are caused by the rupture of an atherosclerotic plaque., The platelet NLRP3 inflammasome promotes secretion of IL-1β, using a direct NLRP3 inhibitor there is a reduction of human platelet aggregation [74]. The first-line therapy is to reduce LDL levels by using statins [75]. There are studies that support the important role by ROS in mechanism of platelet activation and this could be reduced by therapy antioxidant trough HDLs that effects on platelets that can interfere with thrombus formation by blocking their activation and aggregation [76,77,78], preventing degranulation, and altering the spreading as well as their adhesion to fibrinogen. Also, HDL inhibit thrombin-induced tissue factor expression in endothelial cells isolated from humans [79,80], increase endothelial tissue factor pathway inhibitor (TFPI) [81], and may interfere in the production of platelets from bone marrow megakaryocyte progenitor cells [82,83,84]. Epidemiological studies showed that HDL-C is associated with an increased risk of atherothrombotic disease [85,86]. In models testing shear stress, the HDL/Apo-A1 complex has antiadhesive and antithrombotic properties unrelated to RCT [87]. 

### 1.5. HDL Dysfunctional

The Framingham Heart Study [77] established that HDL levels under 35 mg/dL can lead to the development of a cardiovascular event while levels of 60 mg/dL are protective. Thus, the target of various therapies has been to increase the HDL levels, thereby decreasing the cardiovascular damage in patients [9,76,77,88]. 

The serum concentration of cholesterol HDL (C-HDL) is only one of the markers of HDL particle number and has a feeble correlation with HDL functions. Multiple pathologic conditions, such as rheumatoid arthritis [89], hyperuricemia [90,91], diabetes mellitus [92], and acute coronary syndrome [93] trigger structural and functional alterations in HDL, becoming proinflammatory molecules. The above confirms that the function of HDL depends on its composition, such that alterations in its constituent enzymes and proteins result in changes in activity in conditions of oxidative stress, infection, and inflammation [78,94].

Some researchers have used the term “dysfunctional HDL” to describe the changes in their composition and their functional properties. Therefore, alterations in various structural components lead to a state of dysfunction independently of their serum concentration [17]. Asztalos and Schaefer have described that patients who have suffered a coronary event show dysfunctional HDL particles, despite having high C-HDL levels. These patients have a decreased transport of cholesterol to the liver, impaired anti-oxidative effects, a reduced ability to inhibit adhesion molecule expression on endothelial cells, and a reduced synthesis of endothelial nitric oxide [88]. The adverse effects of dysfunctional HDL may explain how, despite having high HDL plasma levels, the specific composition of HDL can contribute to the rupture of atherosclerotic plaque and myocardial infarction [95].

Han et al. [96] showed that HDL particles are also markedly altered in atherosclerosis and are enriched in inflammatory proteins such as apoCIII and serum amyloid A (SAA). HDL enriched with SAA impairs cellular cholesterol efflux and impairs the anti-inflammatory properties of HDL [96]. Schwertani et al. [97] recently demonstrated that desmocollin 1 (DSC1) acts as an apolipoprotein A-I binding protein that is highly expressed in atherosclerotic plaques and inhibits atheroprotective HDL functions by retaining apoA-I. Concentrations of phospolipids, unsaturated fatty acids, omega-3 fatty acids, and sphingomyelin appear to be significantly higher in earlier stages of CVD than in more severe cases. Patients with CVD and Type 2 diabetes mellitus (DM2) present other changes in the lipid composition of HDL, including higher triglycerides, saturated fatty acids, diallyl fatty acids, linoleic acid, Lysophosphatidylcholine (LPC), palmitate-rich triglycerides, and diglycerides [98].

On the other hand, the myeloperoxidase enzyme is a pro-inflammatory enzyme that induces both oxidative modification and nitrosylation of specific residues on plasma and arterial apolipoprotein A-I. It renders dysfunctional HDL particles, which results in impaired member 1 of human transporter sub-family ABCA (ABCA1) macrophage transport, the activation of inflammatory pathways, and an increased risk of coronary artery disease. Therefore, it is of supreme importance to research the biological activity of HDL, both in quantity and quality, as well as to reconsider the basic steps of HDL biogenesis [98,99].

### 1.6. Pharmacological therapies

For many years, it has been suggested that pharmacological therapies (Table 1) can modulate lipid metabolism and will affect HDL-C and triglyceride levels, decreasing the risk of developing cardiovascular disease and myocardial infarction. A meta-analysis made with 15,252 individuals showed a 2–3% reduction in cardiovascular disease when plasma HDL-C was increased to 1 mg/dL [9]. These results stimulated interest in pharmacological treatments to control HDL levels. New generation drugs have been designed to increase HDL, and their functional activity is promising in the significant reversion of atherosclerotic plaques. Nevertheless, these treatments have not been successful in clinical trials or are ineffective for ameliorated risk factors such as hypertension or hyperlipidemia [100,101]. 

Three drugs have been used to increase HDL levels, and to decrease cardiovascular risk and dyslipidemia (abnormal amount of lipids in the blood): fibrates, statins, and niacin. Fibrates increase HDL levels by approximately 10% but seem to have an unfavorable effect on the large HDL subclasses; it has been proposed that fibrates have a cardioprotective function, not by increasing the concentration of HDL, but by an unknown mechanism [100,101]. Statins also increase HDL levels, especially the large subclasses. Further, the changes in HDL are associated with a decreased CETP activity so that the transport of cholesteryl esters and triglycerides between the lipoproteins is decreased [111,112]. In clinical trials, the use of statins in patients with a high cardiovascular risk significantly reduced the occurrence of cardiovascular events in almost half the patients. On the other hand, niacin increases the levels of HDL (mainly large HDL subclasses such as HDL2b-HDL2a) [113,114], and decreases LDL-C and lipoprotein (a) [115]. Niacin has been tested in clinical studies as a coadjuvant to statin treatment which can reduce total cholesterol and VLDL-C and is related to the attenuation of biomarkers of inflammation [116]. Although this therapy has been used clinically for several decades to decrease cholesterol levels, its mechanism of action is not well understood. However, despite the increase in HDL-C and decrease of LDL-C levels, a little more than half of the patients still suffered cardiovascular events [114].

### 1.7. Recent Strategies for Improving HDL Function

Some therapies have been developed in recent years by improving the metabolism, structure, and function of HDL. Some of these therapies utilize Apo-A1 mimetic peptides [117], liver receptor X (LXR) agonists [118], farnesoid X-receptor agonists (FXR) [119], microRNAs (miRNAs) [120], and antisense oligonucleotides targeting genes that are implicated in HDL metabolism, mostly affecting the *CETP* and *APOC3* genes. Apo-A1 mimetic peptides are small amphipathic peptides synthesized to be similar to Apo-A1 in structure and biological activity. They may have a beneficial impact on the metabolism and biological activities of HDL [121] and increase the reverse transport of cholesterol, which has resulted in decreased atherosclerosis in animal models [68,117]. However, these peptides are limited by their low bioavailability, short half-life, and instability in the digestive system [117,121,122].

The natural and synthetic LXR agonists induce the transcriptional activity of LXR target genes, increasing cholesterol catabolism and reducing atherosclerosis in mice [123]. However, these anti-atherogenic effects are not reproduced in CETP-expressing species, including humans, and instead, fatty acid number, triglyceride, and LDL-C levels increase, which leads to hepatic steatosis [118].

FXR agonists are bile acid-activated transcription factors which induce the transcription of transporter genes involved in promoting bile acid clearance and repress genes involved in bile acid biosynthesis. In humans, the protection by the synthetic FXR agonist occurs via the induction of HDL-mediated cholesterol excretion [124].

Because miRNAs (small non-coding RNA molecules) can post-transcriptionally regulate gene expression, they have been employed as a strategy to control cholesterol homeostasis [125]. Indeed, miR-33 increases HDL levels and seems to be a key autophagy regulator and effector in macrophages to generate free cholesterol for efflux with anti-atherosclerotic effects [120,124,125]. Despite the efforts to increase plasma HDL levels or activity of the components of HDL, the death rate from cardiovascular diseases continues to be high. In many cases, the diverse treatments in animals and clinical cases do not have the same effects which makes it difficult to stablish the risk factors, thus, the prevalence of cardiovascular risk remains high [126,127].

PON1 is synthesized in the liver of mammals. Studies suggest that high PON1 activity is associated with the reduction of atherosclerosis and low PON1 levels are considered an independent risk factor for coronary events and other metabolic diseases [128,129,130]. Two related proteins, PON2 and PON3, also have antioxidant activity [131]. PON circulates in the blood bound to Apo-A1 and Apo J of HDL. *PON1* expression is inhibited by proatherogenic conditions and is altered by environmental factors including diet, physical activity, certain drugs, and genetic factors [132]. PON1 has been studied for its possible role in preserving lipoprotein integrity and its antioxidant protective activity of HDL [133,134,135]. PON1 can neutralize hydrogen peroxide and free peroxidized lipids from atherosclerotic lesions [136].

Despite the strong relationship of PON to HDL, HDL concentration and activity of the enzyme do not always correlate [137]. A variety of non-genetic factors have been shown to influence the activity and serum PON1 levels. Under oxidative stress conditions, PON1 activity decreases significantly. This decrease can be compensated by dietary antioxidants such as polyphenols or other bioactive compounds, probably preserving the enzyme integrity. Moderate consumption of alcohol and vitamins C and E also increase serum levels of PON1 in humans and animals [138,139,140] (Table 2).

Specific dietary interventions show promise in reducing atherosclerosis by several mechanisms directly and indirectly related to HDL. Foods, especially fruits, may have high contents of polyphenols, which act as antioxidants by donating a hydrogen atom to negatively charged free radicals [143,144]. Regular consumption of polyphenols, in particular, from pomegranate fruit, increases the expression of the PON1 mRNA in hepatocytes [128] and has anti-atherogenic effects. The progression of atherosclerosis was inhibited in LDL receptor-deficient mice supplemented with quercetin (an antioxidant), which is found in pomegranates, and increased hepatic expression of the *PON1* gene as well as the associated activity of the enzyme in serum [140]. Mice fed an atherogenic (high fat) diet supplemented with fresh pomegranate juice showed an increase in *PON1* gene expression and activity, in addition to an increase in the plasma concentration of HDL [128]. Furthermore, regular consumption of pomegranate juice has been shown to reduce atherogenic LDL alterations in Apo E deficient animals [145], and to prevent endothelial dysfunction due to the activation of the serine/threonine-protein kinase/Endothelial nitric oxide synthase (Akt/eNOS) pathway, possibly mediated by HDL through its molecular component sphingosine-1 phosphate [146].

### 1.8. Endothelial Dysfunction and Exogenous Antioxidants

The endothelium is a monolayer of cells that coat the lumen of blood vessels. Endothelial cells are connected by intercellular junctions that restrict the traffic of macromolecules between the blood and the vascular wall [147]. The endothelium may perform anti-thrombotic functions such as inhibition of platelet adhesion and coagulation, regulation of the fibrinolytic system, and control of muscle tone and cell proliferation through monitoring the activity of smooth muscle cells. The endothelium also modulates the transit of macromolecules such as lipoproteins and controls cell adhesion of leukocytes to the arterial wall [148].

Nitric oxide (NO) is a water-soluble gas continuously synthesized from the amino acid arginine in endothelial cells, which is known to have vascular functions as a vasodilator and second messenger in the vascular wall. HDL stimulates the production of NO, partially elevating levels of sphingosine 1-phosphate (S1P) that might facilitate interaction with S1P receptors and indirectly ensuing NO generation [149,150]. Endothelial cells cultured in high atherogenic LDL-C concentrations (>160 mg/dL) improves the metabolism of arachidonic acid, which acts as a second messenger regulating many cellular processes and reduces the production of NO. The loss of NO results in upregulation of cell adhesion molecules (CAM); thus, CAM upregulation renders the endothelium sticky [151]. On the other hand, HDL promotes vascular endothelial function by inducing an anti-inflammatory mechanism reducing the expression of the cell adhesion molecules ICAM1, VCAM1, and selectins.

Endothelial dysfunction is an imbalance in the bioavailability of active substances of endothelial origin predisposing the vasculature to inflammation, vasoconstriction, and increased permeability which facilitates the development of atherosclerosis, platelet aggregation, and thrombosis. Endothelial dysfunction is one of the first manifestations of both hypertension and atherosclerosis [152]. An increased plasma LDL concentration is one of the factors associated with a modification in endothelial function by decreasing endothelium-dependent vasodilation [153] and increasing the level of adhesion molecules [154]. One of the most studied functions of HDL is their interaction with the ABCA1 membrane protein that drives the cholesterol efflux system [155]. HDL is also reported to transport proteins, vitamins, hormones, and microRNAs to different recipient organs. These HDLs have exclusive characteristics that allow them to deliver cargo to specific targets, suggesting that these lipoproteins play a multi-faceted role in intracellular communication [156]. Some sterols of dietary origin (particularly plant sterols and hydrophobic antioxidants such as vitamin E, lutein, and zeaxanthin) are absorbed in the intestines in a process that involves the HDL formation pathway; lutein and zeaxanthin are carotenoids with, as well as some polyphenols, potential anti-inflammatory properties, and their absorption could improve the beneficial HDL properties [157,158,159].

Some experimental evidence indicates that fruits containing polyphenols modulate risk factors in the development of cardiovascular diseases [160,161], and the consumption of polyphenol vitamin supplements and polyphenol-containing foods may prevent cardiovascular diseases [162,163]. Pomegranate has been shown to be especially effective in reducing risk factors. The consumption of polyphenol-containing pomegranate juice resulted in an improvement of stress-induced myocardial ischemia in patients with coronary artery disease [145], and the reduction of atherosclerotic lesions in patients with carotid artery stenosis [164]. Administration of pomegranate extracts to animals with hypercholesterolemia-induced coronary dysfunction increased their endothelium-dependent vasodilation and induced NO-mediated relaxation through a mechanism involving the endothelial activation of Akt [165].

Polyphenols can be more effective as cardioprotective agents relative to some vitamins and carotenes (Table 3). Studies are needed to compare individual with multiple polyphenols, determine the effectiveness of polyphenols as purified molecules relative to polyphenols in the native state in foods, and discover if other compounds in foods are also important in the activity of polyphenols. The different components of lipid profiles such as total cholesterol levels, HDL-C concentration [166], lipid peroxidation [167], triglycerides and LDL-C [168], that are affected by polyphenols, and the specific improvements of vascular function and cardiovascular risk factors induced by these compounds also needs to be studied further.

## 2. Conclusions

The various therapies for the improvement of HDL have not decreased the prevalence of cardiovascular diseases; thus, cardiovascular disease is probably due to other factors than HDL such as disequilibrium in endothelial function. A possible explanation is that several metabolic or signaling pathways act together and the new therapies have not been able to modify all the necessary pathways. Recent studies show that the consumption of food rich in polyphenols as an alternative intervention may be more effective in reducing cardiovascular risk, possibly to a change in the biochemical HDL composition. At this point, it is necessary to focus on the development of new methodologies to understand the change in biochemical composition in nutritional trials. However, well-controlled clinical trials are necessary to discover the molecular and biochemical mechanisms affected to use them as therapies for the reduction of coronary events.

## Figures and Tables

**Table 1:** Pharmacological treatments for decreasing the risk of cardiovascular disease.

Drug	Effect of Drugs on HDL-C and Cardiovascular Health	Author
Statins	Women and men (32,258) obtained from the (an individual patient data meta-analysis of statin therapY in at risk Groups:	
	Effects of rosuvastatin, atorvastatin and simvastatin) VOYAGER study who received atorvastatin (10–80 mg), rosuvastatin (5–40 mg) or simvastatin (10–80 mg); all statins and doses decreased the concentration of LDL-C and increased the HDL-C.	[102]
	A meta-analysis of genome-wide association in a population of European descendents was made to identify variants that modify HDL-C. Participants (27,720) showed an association between the cholesterol ester transporter protein CETP locus (chromosome 16) and HDL-C response to statin treatment.	[103]
Nicotinic acid	Obese, nondiabetic, hypertriglyceridemic males (19) with low HDL-C levels received nicotinic acid for eight weeks, achieving a decrease in biomarkers of inflammation, cell adhesion and cell proliferation in addition to LDL-C and total cholesterol.	[104]
	AIM-HIGH individuals (2457) with cardiovascular disease at baseline and one year of treatment of extended-release niacin and high triglycerides (>200 mg/dL) and very low HDL-C (<32 mg/dL) showed a significant reduction in serum levels of remnant lipoprotein cholesterol and increased HDL2-C.	[105]
Fibrates	Participants of a Cochrane Collaboration study (16,112) showed a protective effect of fibrates and safety in the secondary prevention of different cardiovascular events including coronary and cerebrovascular disease.	[106]
	In individuals with hypertriglyceridemia (7389) and 5068 individuals with hypertriglyceridemia and low HDL-C levels, the treatment with fibrates reduced the subsequent vascular event risk.	[107]
Bile acid binding resins	Patients treated with ezetimibe (10 mg/day) (302), 1234 patients treated with simvastatin (10 mg/day, 20 mg/day, 40 mg/day or 80 mg/day) and 1236 patients with combination of both drugs (10/10 mg/day, 10/20 mg/day, 10/40 mg/day or 10/80 mg/day), are associated with smaller decreases in Apo B compared with LDL-C and non-HDL-C.	[108]
CETP inhibitors	Patients with increased HDL-C (2826), and 3739 patients with reduced triglyceride levels and LDL-C showed an increase in blood pressure due to the increase of LDL metabolism through their receptors. Different CETP inhibitors (mainly anacetrapib) are used to increase HDL-C and Apo-A1 levels and significantly alter HDL2 subclasses and pre-β HDL particles with a decrease in LDL-C.	[109,110]

**Table 2:** Proteins associated with HDL3. PAF-AH = platelet-activating factor acetylhydrolase.

Proteins Interacting with HDL3	Effects on HDL3	Reference
PON1	PON1 increases macrophage cholesterol efflux and improves the antioxidant properties of HDL.	[71]
PAF-AH	PAF-AH activity and expression are upregulated by mediators of inflammation at the transcriptional level; their stability provides antioxidant properties and anti-atherogenic activities to HDL3.	[72,141]
LCAT	Supplementation of the enzyme LCAT is a potential therapeutic intervention for HDL abnormalities that result from specific mutations on Apo-A1.	[142]

**Table 3:** Nutrients with cardioprotective properties.

Nutrients	Natural Sources	Mechanism
Vitamin B3	Chicken, fish, peanuts, and legumes	Involved in catabolism and synthesis of HDL; increase the production of Apo-A1 and expression of ABCA1; helps to transfer the cholesterol from macrophages to nascent HDL; decreases the expression and activity of CETP [169,170].
Omega 3 fatty acids	Salmon, peas, tuna, sardines, and trout	Reduces the uptake and binding of LDL to the arterial wall due to a reduction of lipoprotein-lipase levels and macrophages; facilitates the incorporation of omega-3 into the phospholipid membrane; changes arachidonic acid metabolism reducing the release of thromboxane A2 [171,172].
Vitamin E (α-, β-, γ-, δ-tocopherol and tocotrienol)	Wheat germ oil, sunflower seeds, almonds, peanuts, corn oil, olive oil, spinach, broccoli, soybean oil, kiwi, mango and tomato	Reduces the expression of VCAM-1, ICAM-1 and e-selectin; decreases the adhesion of leukocytes into the endothelium or arterial wall [173,174].
Lycopene	Tomatoes, grapefruit, watermelon, and papaya	Reduces intima wall thickness or lesions in aorta mainly to its antioxidant activity related to LDL oxidation; inhibits the activity and expression of 3-hydroxy-methyl glutaryl (HMG)-CoA, reducing cholesterol synthesis [175].
Gallic acid, punicalagin	Pomegranate juice	Reduces the expression of inflammatory cytokines TNF-α, IL-1β, IL-18 and nuclear factor kappa-light-chain-enhancer of activated B cells (NF-κβ) [176]; inhibits LDL oxidation and macrophage foam cell formation due to the accumulation of these polyphenols in arterial macrophages besides inactive ROS and reactive nitrogen species (RNS) [177].

Note: The majority of nutrients do not change the conformation or activity of HDL. However, they might be associated with HDL, improving its function.

## References

[1] Mahley R.W., Innerarity T.L., Rall S.C., Weisgraber K.H. (1984). Plasma lipoproteins: Apolipoprotein structure and function. J. Lipid Res..

[2] Mahley R.W., Ji Z.S. (1999). Remnant lipoprotein metabolism: Key pathways involving cell-surface heparan sulfate proteoglycans and apolipoprotein E. J. Lipid Res..

[3] Segrest J.P., Jones M.K., De Loof H., Dashti N. (2001). Structure of apolipoprotein B-100 in low density lipoproteins. J. Lipid Res..

[4] Green P.H., Glickman R.M., Riley J.W., Quinet E. (1980). Human apolipoprotein A-IV. Intestinal origin and distribution in plasma. J. Clin. Investig..

[5] Stampfer M.J., Krauss R.M., Ma J., Blanche P.J., Holl L.G., Sacks F.M., Hennekens C.H. (1996). A prospective study of triglyceride level, low-density lipoprotein particle diameter, and risk of myocardial infarction. JAMA.

[6] Posadas-Romero C., Posadas-Sánchez R., Juárez-Rojas J.G., Medina-Urrutia A., Jorge-Galarza E., Cardoso-Saldaña G., Caracas-Portilla N., Mendoza-Peŕez E. (2008). High and low density lipoprotein abnormalities in coronary patients with LDL-C at target and uncontrolled HDL-C and triglycerides. Arch. Cardiol. Mex..

[7] Cannon C.P., Braunwald E., McCabe C.H., Rader D.J., Rouleau J.L., Belder R., Joyal S.V., Hill K.A., Pfeffer M.A., Skene A.M. (2004). Intensive versus moderate lipid lowering with statins after acute coronary syndromes. N. Engl. J. Med..

[8] LaRosa J.C., Deedwania P.C., Shepherd J., Wenger N.K., Greten H., DeMicco D.A., Breazna A., TNT Investigators (2010). Comparison of 80 versus 10 mg of atorvastatin on occurrence of cardiovascular events after the first event (from the Treating to New Targets [TNT] trial). Am. J. Cardiol..

[9] Gordon D.J., Probstfield J.L., Garrison R.J., Neaton J.D., Castelli W.P., Knoke J.D., Jacobs D.R., Bangdiwala S., Tyroler H.A. (1989). High-density lipoprotein cholesterol and cardiovascular disease. Four prospective American studies. Circulation.

[10] Hokanson J.E., Austin M.A. (1996). Plasma triglyceride level is a risk factor for cardiovascular disease independent of high-density lipoprotein cholesterol level: A meta-analysis of population-based prospective studies. J. Cardiovasc. Risk.

[11] Stahel P., Xiao C., Hegele R.A., Lewis G.F. (2018). The Atherogenic Dyslipidemia Complex and Novel Approaches to Cardiovascular Disease Prevention in Diabetes. Can. J. Cardiol..

[12] Murphy A.J., Woollard K.J., Hoang A., Mukhamedova N., Stirzaker R.A., McCormick S.P.A., Remaley A.T., Sviridov D., Chin-Dusting J. (2008). High-density lipoprotein reduces the human monocyte inflammatory response. Arterioscler. Thromb. Vasc. Biol..

[13] Schaefer E.J., Anthanont P., Diffenderfer M.R., Polisecki E., Asztalos B.F. (2016). Diagnosis and treatment of high density lipoprotein deficiency. Prog. Cardiovasc. Dis..

[14] Calabresi L., Gomaraschi M., Simonelli S., Bernini F., Franceschini G. (2015). HDL and atherosclerosis: Insights from inherited HDL disorders. Biochim. Biophys. Acta.

[15] Paavola T., Kuusisto S., Jauhiainen M., Kakko S., Kangas-Kontio T., Metso J., Soininen P., Ala-Korpela M., Bloigu R., Hannuksela M.L. (2017). Impaired HDL2-mediated cholesterol efflux is associated with metabolic syndrome in families with early onset coronary heart disease and low HDL-cholesterol level. PLoS ONE.

[16] Tan Y., Liu T.R., Hu S.W., Tian D., Li C., Zhong J.K., Sun H.G., Luo T.T., Lai W.Y., Guo Z.-G. (2014). Acute coronary syndrome remodels the protein cargo and functions of high-density lipoprotein subfractions. PLoS ONE.

[17] Tiozzo E., Gardener H., Hudson B.I., Dong C., Della-Morte D., Crisby M., Goldberg R.B., Elkind M.S.V., Cheung Y.K., Wright C.B. (2016). Subfractions of High-Density Lipoprotein-Cholesterol and Carotid Intima-Media Thickness. Stroke.

[18] Martin S.S., Khokhar A.A., May H.T., Kulkarni K.R., Blaha M.J., Joshi P.H., Toth P.P., Muhlestein J.B., Anderson J.L., Knight S. (2015). Lipoprotein Investigators Collaborative (LIC) HDL cholesterol subclasses, myocardial infarction, and mortality in secondary prevention: The Lipoprotein Investigators Collaborative. Eur. Heart J..

[19] Joshi P.H., Toth P.P., Lirette S.T., Griswold M.E., Massaro J.M., Martin S.S., Blaha M.J., Kulkarni K.R., Khokhar A.A., Correa A. (2016). Lipoprotein Investigators Collaborative (LIC) Study Group Association of high-density lipoprotein subclasses and incident coronary heart disease: The Jackson Heart and Framingham Offspring Cohort Studies. Eur. J. Prev. Cardiol..

[20] Warnick G.R., Nauck M., Rifai N. (2001). Evolution of methods for measurement of HDL-cholesterol: From ultracentrifugation to homogeneous assays. Clin. Chem..

[21] Usui S., Nakamura M., Jitsukata K., Nara M., Hosaki S., Okazaki M. (2000). Assessment of between-instrument variations in a HPLC method for serum lipoproteins and its traceability to reference methods for total cholesterol and HDL-cholesterol. Clin. Chem..

[22] Jiménez B., Holmes E., Heude C., Tolson R.F., Harvey N., Lodge S.L., Chetwynd A.J., Cannet C., Fang F., Pearce J.T.M. (2018). Quantitative Lipoprotein Subclass and Low Molecular Weight Metabolite Analysis in Human Serum and Plasma by 1H NMR Spectroscopy in a Multilaboratory Trial. Anal. Chem..

[23] Rader D.J., Hovingh G.K. (2014). HDL and cardiovascular disease. Lancet Lond. Engl..

[24] Robertson J., Peters M.J., McInnes I.B., Sattar N. (2013). Changes in lipid levels with inflammation and therapy in RA: A maturing paradigm. Nat. Rev. Rheumatol..

[25] Andersen L.B., Riddoch C., Kriemler S., Hills A.P., Hills A. (2011). Physical activity and cardiovascular risk factors in children. Br. J. Sports Med..

[26] Ramirez A., Hu P.P. (2015). Low High-Density Lipoprotein and Risk of Myocardial Infarction. Clin. Med. Insights Cardiol..

[27] Superko H.R., Pendyala L., Williams P.T., Momary K.M., King S.B., Garrett B.C. (2012). High-density lipoprotein subclasses and their relationship to cardiovascular disease. J. Clin. Lipidol..

[28] Hansel B., Giral P., Nobecourt E., Chantepie S., Bruckert E., Chapman M.J., Kontush A. (2004). Metabolic syndrome is associated with elevated oxidative stress and dysfunctional dense high-density lipoprotein particles displaying impaired antioxidative activity. J. Clin. Endocrinol. Metab..

[29] Kontush A., de Faria E.C., Chantepie S., Chapman M.J. (2005). A normotriglyceridemic, low HDL-cholesterol phenotype is characterised by elevated oxidative stress and HDL particles with attenuated antioxidative activity. Atherosclerosis.

[30] Errico T.L., Chen X., Martin Campos J.M., Julve J., Escolà-Gil J.C., Blanco-Vaca F. (2013). Basic mechanisms: Structure, function and metabolism of plasma lipoproteins. Clin. Investig. Arterioscler. Publ. Soc. Esp. Arterioscler..

[31] Fielding C.J., Fielding P.E. (1982). Cholesterol transport between cells and body fluids. Role of plasma lipoproteins and the plasma cholesterol esterification system. Med. Clin. N. Am..

[32] Vladimirov S., Gojkovic T., Spasojevic-Kalimanovska V., Zeljkovic A., Vekic J., Kalimanovska-Ostric D., Jelic-Ivanovic Z. (2017). Influence of LCAT and CETP activity on the reverse cholesterol transport and modification of HDL particles in statin-treated coronary artery disease patients and healthy subjects. Atherosclerosis.

[33] Norum K.R. (2017). The function of lecithin:cholesterol acyltransferase (LCAT). Scand. J. Clin. Lab. Investig..

[34] Redondo S., Martínez-González J., Urraca C., Tejerina T. (2011). Emerging therapeutic strategies to enhance HDL function. Lipids Health Dis..

[35] Lewis G.F. (2006). Determinants of plasma HDL concentrations and reverse cholesterol transport. Curr. Opin. Cardiol..

[36] Cuchel M., Lund-Katz S., de la Llera-Moya M., Millar J.S., Chang D., Fuki I., Rothblat G.H., Phillips M.C., Rader D.J. (2010). Pathways by which reconstituted high-density lipoprotein mobilizes free cholesterol from whole body and from macrophages. Arterioscler. Thromb. Vasc. Biol..

[37] Julve J., Llaverias G., Blanco-Vaca F., Escolà-Gil J.C. (2011). Seeking novel targets for improving in vivo macrophage-specific reverse cholesterol transport: Translating basic science into new therapies for the prevention and treatment of atherosclerosis. Curr. Vasc. Pharmacol..

[38] Cordero A., Moreno-Arribas J., Bertomeu-González V., Agudo P., Miralles B., Masiá M.D., López-Palop R., Bertomeu-Martínez V. (2012). Low levels of high-density lipoproteins cholesterol are independently associated with acute coronary heart disease in patients hospitalized for chest pain. Rev. Esp. Cardiol. Engl. Ed..

[39] Fan J., Qi Y., Zhao D. (2014). A meta-analysis on the association between high-density lipoprotein particle subfractions and cardiovascular disease events. Zhonghua Xin Xue Guan Bing Za Zhi.

[40] Boekholdt S.M., Arsenault B.J., Hovingh G.K., Mora S., Pedersen T.R., Larosa J.C., Welch K.M.A., Amarenco P., Demicco D.A., Tonkin A.M. (2013). Levels and changes of HDL cholesterol and apolipoprotein A-I in relation to risk of cardiovascular events among statin-treated patients: A meta-analysis. Circulation.

[41] Kaur N., Pandey A., Negi H., Shafiq N., Reddy S., Kaur H., Chadha N., Malhotra S. (2014). Effect of HDL-raising drugs on cardiovascular outcomes: A systematic review and meta-regression. PLoS ONE.

[42] Goff D.C., Lloyd-Jones D.M., Bennett G., Coady S., D’Agostino R.B., Gibbons R., Greenland P., Lackland D.T., Levy D., O’Donnell C.J. (2014). American College of Cardiology/American Heart Association Task Force on Practice Guidelines 2013 ACC/AHA guideline on the assessment of cardiovascular risk: A report of the American College of Cardiology/American Heart Association Task Force on Practice Guidelines. J. Am. Coll. Cardiol..

[43] Ibanez B., Vilahur G., Cimmino G., Speidl W.S., Pinero A., Choi B.G., Zafar M.U., Santos-Gallego C.G., Krause B., Badimon L. (2008). Rapid change in plaque size, composition, and molecular footprint after recombinant apolipoprotein A-I Milano (ETC-216) administration: Magnetic resonance imaging study in an experimental model of atherosclerosis. J. Am. Coll. Cardiol..

[44] Toth P.P., Barter P.J., Rosenson R.S., Boden W.E., Chapman M.J., Cuchel M., D’Agostino R.B., Davidson M.H., Davidson W.S., Heinecke J.W. (2013). High-density lipoproteins: A consensus statement from the National Lipid Association. J. Clin. Lipidol..

[45] Bohr A.-H., Pedersen F.K., Nielsen C.H., Müller K.G. (2016). Lipoprotein cholesterol fractions are related to markers of inflammation in children and adolescents with juvenile idiopathic arthritis: A cross sectional study. Pediatr. Rheumatol. Online J..

[46] Avina-Zubieta J.A., Thomas J., Sadatsafavi M., Lehman A.J., Lacaille D. (2012). Risk of incident cardiovascular events in patients with rheumatoid arthritis: A meta-analysis of observational studies. Ann. Rheum. Dis..

[47] Millar C.L., Duclos Q., Blesso C.N. (2017). Effects of Dietary Flavonoids on Reverse Cholesterol Transport, HDL Metabolism, and HDL Function. Adv. Nutr..

[48] Namiri-Kalantari R., Gao F., Chattopadhyay A., Wheeler A.A., Navab K.D., Farias-Eisner R., Reddy S.T. (2015). The dual nature of HDL: Anti-Inflammatory and pro-Inflammatory. BioFactors Oxf. Engl..

[49] Dodani S., Kaur R., Reddy S., Reed G.L., Navab M., George V. (2008). Can dysfunctional HDL explain high coronary artery disease risk in South Asians?. Int. J. Cardiol..

[50] Roberts C.K., Ng C., Hama S., Eliseo A.J., Barnard R.J. (2006). Effect of a short-term diet and exercise intervention on inflammatory/anti-inflammatory properties of HDL in overweight/obese men with cardiovascular risk factors. J. Appl. Physiol. 1985.

[51] Perségol L., Vergès B., Gambert P., Duvillard L. (2007). Inability of HDL from abdominally obese subjects to counteract the inhibitory effect of oxidized LDL on vasorelaxation. J. Lipid Res..

[52] Lee J.M.S., Robson M.D., Yu L.-M., Shirodaria C.C., Cunnington C., Kylintireas I., Digby J.E., Bannister T., Handa A., Wiesmann F. (2009). Effects of high-dose modified-release nicotinic acid on atherosclerosis and vascular function: A randomized, placebo-controlled, magnetic resonance imaging study. J. Am. Coll. Cardiol..

[53] Moriyama K., Negami M., Takahashi E. (2014). HDL2-cholesterol/HDL3-cholesterol ratio was associated with insulin resistance, high-molecular-weight adiponectin, and components for metabolic syndrome in Japanese. Diabetes Res. Clin. Pract..

[54] Arts E., Fransen J., Lemmers H., Stalenhoef A., Joosten L., van Riel P., Popa C.D. (2012). High-density lipoprotein cholesterol subfractions HDL2 and HDL3 are reduced in women with rheumatoid arthritis and may augment the cardiovascular risk of women with RA: A cross-sectional study. Arthritis Res. Ther..

[55] Brites F.D., Bonavita C.D., De Geitere C., Cloës M., Delfly B., Yael M.J., Fruchart J., Wikinski R.W., Castro G.R. (2000). Alterations in the main steps of reverse cholesterol transport in male patients with primary hypertriglyceridemia and low HDL-cholesterol levels. Atherosclerosis.

[56] Stampfer M.J., Sacks F.M., Salvini S., Willett W.C., Hennekens C.H. (1991). A prospective study of cholesterol, apolipoproteins, and the risk of myocardial infarction. N. Engl. J. Med..

[57] Di Angelantonio E., Gao P., Pennells L., Kaptoge S., Caslake M., Thompson A., Butterworth A.S., Sarwar N., Wormser D., Saleheen D. (2012). Lipid-related markers and cardiovascular disease prediction. JAMA.

[58] Witztum J.L., Steinberg D. (2001). The oxidative modification hypothesis of atherosclerosis: Does it hold for humans?. Trends Cardiovasc. Med..

[59] Fateeva V.V. (2017). Pathogenesis of endothelial dysfunction in cerebral atherosclerosis and their correction. Zh Nevrol Psikhiatr Im S S Korsakova.

[60] Tawakol A., Jaffer F. (2018). Imaging the Intersection of Oxidative Stress, Lipids, and Inflammation: Progress Toward Personalized Care of Atherosclerosis. J. Am. Coll. Cardiol..

[61] Ooi B.K., Goh B.H., Yap W.H. (2017). Oxidative Stress in Cardiovascular Diseases: Involvement of Nrf2 Antioxidant Redox Signaling in Macrophage Foam Cells Formation. Int. J. Mol. Sci..

[62] Varadharaj S., Kelly O.J., Khayat R.N., Kumar P.S., Ahmed N., Zweier J.L. (2017). Role of Dietary Antioxidants in the Preservation of Vascular Function and the Modulation of Health and Disease. Front. Cardiovasc. Med..

[63] Ibitoye O.B., Ajiboye T.O. (2018). Dietary phenolic acids reverse insulin resistance, hyperglycaemia, dyslipidaemia, inflammation and oxidative stress in high-fructose diet-induced metabolic syndrome rats. Arch. Physiol. Biochem..

[64] Huyut Z., Beydemir Ş., Gülçin İ. (2017). Antioxidant and Antiradical Properties of Selected Flavonoids and Phenolic Compounds. Biochem. Res. Int..

[65] Griffiths K., Aggarwal B.B., Singh R.B., Buttar H.S., Wilson D., De Meester F. (2016). Food Antioxidants and Their Anti-Inflammatory Properties: A Potential Role in Cardiovascular Diseases and Cancer Prevention. Diseases.

[66] Chistiakov D.A., Melnichenko A.A., Orekhov A.N., Bobryshev Y.V. (2017). Paraoxonase and atherosclerosis-related cardiovascular diseases. Biochimie.

[67] Gaut J.P., Heinecke J.W. (2001). Mechanisms for oxidizing low-density lipoprotein. Insights from patterns of oxidation products in the artery wall and from mouse models of atherosclerosis. Trends Cardiovasc. Med..

[68] Song X., Fischer P., Chen X., Burton C., Wang J. (2009). An apoA-I mimetic peptide facilitates off-loading cholesterol from HDL to liver cells through scavenger receptor BI. Int. J. Biol. Sci..

[69] Miyata M., Smith J.D. (1996). Apolipoprotein E allele-specific antioxidant activity and effects on cytotoxicity by oxidative insults and beta-amyloid peptides. Nat. Genet..

[70] Kelso G.J., Stuart W.D., Richter R.J., Furlong C.E., Jordan-Starck T.C., Harmony J.A. (1994). Apolipoprotein J is associated with paraoxonase in human plasma. Biochemistry.

[71] Rosenblat M., Volkova N., Aviram M. (2014). HDL3 stimulates paraoxonase 1 antiatherogenic catalytic and biological activities in a macrophage model system: In vivo and in vitro studies. BioFactors Oxf. Engl..

[72] Stafforini D.M. (2015). Plasma PAF-AH (PLA2G7). Enzymes.

[73] Samad F., Ruf W. (2013). Inflammation, obesity, and thrombosis. Blood.

[74] Qiao J., Wu X., Luo Q., Wei G., Xu M., Wu Y., Liu Y., Li X., Zi J., Ju W. (2018). NLRP3 regulates platelet integrin αIIbβ3 outside-in signaling, hemostasis and arterial thrombosis. Haematologica.

[75] Annema W., von Eckardstein A., Kovanen P.T. (2015). HDL and Atherothrombotic Vascular Disease.

[76] Wilson P.W., D’Agostino R.B., Levy D., Belanger A.M., Silbershatz H., Kannel W.B. (1998). Prediction of coronary heart disease using risk factor categories. Circulation.

[77] Castelli W.P., Anderson K., Wilson P.W.F., Levy D. (1992). Lipids and risk of coronary heart disease The Framingham Study. Ann. Epidemiol..

[78] Sammalkorpi K., Valtonen V., Kerttula Y., Nikkilä E., Taskinen M.R. (1988). Changes in serum lipoprotein pattern induced by acute infections. Metabolism.

[79] Viswambharan H., Ming X.-F., Zhu S., Hubsch A., Lerch P., Vergères G., Rusconi S., Yang Z. (2004). Reconstituted High-Density Lipoprotein Inhibits Thrombin-Induced Endothelial Tissue Factor Expression Through Inhibition of RhoA and Stimulation of Phosphatidylinositol 3-Kinase but not Akt/Endothelial Nitric Oxide Synthase. Circ. Res..

[80] Griffin J.H., Kojima K., Banka C.L., Curtiss L.K., Fernández J.A. (1999). High-density lipoprotein enhancement of anticoagulant activities of plasma protein S and activated protein C. J. Clin. Investig..

[81] Holy E., Besler C., Reiner M., Camici G., Manz J., Beer J., Lüscher T., Landmesser U., Tanner F. (2014). High-density lipoprotein from patients with coronary heart disease loses anti-thrombotic effects on endothelial cells: Impact on arterial thrombus formation. Thromb. Haemost..

[82] Calkin A.C., Drew B.G., Ono A., Duffy S.J., Gordon M.V., Schoenwaelder S.M., Sviridov D., Cooper M.E., Kingwell B.A., Jackson S.P. (2009). Reconstituted High-Density Lipoprotein Attenuates Platelet Function in Individuals with Type 2 Diabetes Mellitus by Promoting Cholesterol Efflux. Circulation.

[83] Badrnya S., Assinger A., Volf I. (2013). Native High Density Lipoproteins (HDL) Interfere with Platelet Activation Induced by Oxidized Low Density Lipoproteins (OxLDL). Int. J. Mol. Sci..

[84] Murphy A.J., Bijl N., Yvan-Charvet L., Welch C.B., Bhagwat N., Reheman A., Wang Y., Shaw J.A., Levine R.L., Ni H. (2013). Cholesterol efflux in megakaryocyte progenitors suppresses platelet production and thrombocytosis. Nat. Med..

[85] Di Angelantonio E., Sarwar N., Perry P., Kaptoge S., Ray K.K., Thompson A., Wood A.M., Lewington S., Sattar N., Packard C.J. (2009). Major Lipids, Apolipoproteins, and Risk of Vascular Disease. JAMA.

[86] Barter P., Gotto A.M., LaRosa J.C., Maroni J., Szarek M., Grundy S.M., Kastelein J.J.P., Bittner V., Fruchart J.-C. (2007). Treating to New Targets Investigators. HDL Cholesterol, Very Low Levels of LDL Cholesterol, and Cardiovascular Events. N. Engl. J. Med..

[87] Federici A.B. (2016). HDL/ApoA-I: Role in VWF-dependent thrombosis. Blood.

[88] Asztalos B.F., Schaefer E.J. (2003). High-density lipoprotein subpopulations in pathologic conditions. Am. J. Cardiol..

[89] Rodríguez-Carrio J., Alperi-López M., López P., López-Mejías R., Alonso-Castro S., Abal F., Ballina-García F.J., González-Gay M.Á., Suárez A. (2017). High triglycerides and low high-density lipoprotein cholesterol lipid profile in rheumatoid arthritis: A potential link among inflammation, oxidative status, and dysfunctional high-density lipoprotein. J. Clin. Lipidol..

[90] Meléndez-Ramírez G., Pérez-Méndez O., López-Osorio C., Kuri-Alfaro J., Espinola-Zavaleta N. (2012). Effect of the Treatment with Allopurinol on the Endothelial Function in Patients with Hyperuricemia. Endocr. Res..

[91] Martínez-Ramírez M., Flores-Castillo C., Sánchez-Lozada L.G., Bautista-Pérez R., Carreón-Torres E., Fragoso J.M., Rodriguez-Pérez J.M., García-Arroyo F.E., López-Olmos V., Luna-Luna M. (2017). Hyperuricemia is Associated with Increased Apo AI Fractional Catabolic Rates and Dysfunctional HDL in New Zealand Rabbits. Lipids.

[92] Srivastava R.A.K. (2018). Dysfunctional HDL in diabetes mellitus and its role in the pathogenesis of cardiovascular disease. Mol. Cell. Biochem..

[93] Carnuta M.G., Stancu C.S., Toma L., Sanda G.M., Niculescu L.S., Deleanu M., Popescu A.C., Popescu M.R., Vlad A., Dimulescu D.R. (2017). Dysfunctional high-density lipoproteins have distinct composition, diminished anti-inflammatory potential and discriminate acute coronary syndrome from stable coronary artery disease patients. Sci. Rep..

[94] Pérez-Méndez Ó., Pacheco H.G., Martínez-Sánchez C., Franco M. (2014). HDL-cholesterol in coronary artery disease risk: Function or structure?. Clin. Chim. Acta.

[95] Vergeer M., Holleboom A.G., Kastelein J.J.P., Kuivenhoven J.A. (2010). The HDL hypothesis: Does high-density lipoprotein protect from atherosclerosis?. J. Lipid Res..

[96] Han C.Y., Tang C., Guevara M.E., Wei H., Wietecha T., Shao B., Subramanian S., Omer M., Wang S., O’Brien K.D. (2016). Serum amyloid A impairs the antiinflammatory properties of HDL. J. Clin. Investig..

[97] Schwertani A., Choi H.Y., Genest J. (2018). HDLs and the pathogenesis of atherosclerosis. Curr. Opin. Cardiol..

[98] Annema W., von Eckardstein A. (2016). Dysfunctional high-density lipoproteins in coronary heart disease: Implications for diagnostics and therapy. Transl. Res..

[99] Rosenson R.S., Brewer H.B., Ansell B.J., Barter P., Chapman M.J., Heinecke J.W., Kontush A., Tall A.R., Webb N.R. (2016). Dysfunctional HDL and atherosclerotic cardiovascular disease. Nat. Rev. Cardiol..

[100] Park J.S., Cha K.S., Lee H.W., Oh J.-H., Choi J.H., Lee H.C., Hong T.J., Jeong M.H., Chae S.C., Kim Y.J. (2018). Predictive and protective role of high-density lipoprotein cholesterol in acute myocardial infarction. Cardiol. J..

[101] Talbot D., Delaney J.A.C., Sandfort V., Herrington D.M., McClelland R.L. (2018). Importance of the lipid-related pathways in the association between statins, mortality, and cardiovascular disease risk: The Multi-Ethnic Study of Atherosclerosis. Pharmacoepidemiol. Drug Saf..

[102] Karlson B.W., Palmer M.K., Nicholls S.J., Barter P.J., Lundman P. (2017). Effects of age, gender and statin dose on lipid levels: Results from the VOYAGER meta-analysis database. Atherosclerosis.

[103] Postmus I., Warren H.R., Trompet S., Arsenault B.J., Avery C.L., Bis J.C., Chasman D.I., de Keyser C.E., Deshmukh H.A., Evans D.S. (2016). Meta-analysis of genome-wide association studies of HDL cholesterol response to statins. J. Med. Genet..

[104] Adiels M., Chapman M.J., Robillard P., Krempf M., Laville M., Borén J. (2018). Niacin Study Group Niacin action in the atherogenic mixed dyslipidemia of metabolic syndrome: Insights from metabolic biomarker profiling and network analysis. J. Clin. Lipidol..

[105] Toth P.P., Jones S.R., Slee A., Fleg J., Marcovina S.M., Lacy M., McBride R., Boden W.E. (2018). Relationship between lipoprotein subfraction cholesterol and residual risk for cardiovascular outcomes: A post hoc analysis of the AIM-HIGH trial. J. Clin. Lipidol..

[106] Wang D., Liu B., Tao W., Hao Z., Liu M. (2015). Fibrates for secondary prevention of cardiovascular disease and stroke. Cochrane Database Syst. Rev..

[107] Lee M., Saver J.L., Towfighi A., Chow J., Ovbiagele B. (2011). Efficacy of fibrates for cardiovascular risk reduction in persons with atherogenic dyslipidemia: A meta-analysis. Atherosclerosis.

[108] Farnier M., Guyton J.R., Jensen E., Polis A.B., Johnson-Levonas A.O., Brudi P. (2013). Effects of ezetimibe, simvastatin and ezetimibe/simvastatin on correlations between apolipoprotein B, LDL cholesterol and non-HDL cholesterol in patients with primary hypercholesterolemia. Atherosclerosis.

[109] Li C., Zhang W., Zhou F., Chen C., Zhou L., Li Y., Liu L., Pei F., Luo H., Hu Z. (2013). Cholesteryl ester transfer protein inhibitors in the treatment of dyslipidemia: A systematic review and meta-analysis. PLoS ONE.

[110] Filippatos T.D., Kei A., Elisaf M.S. (2017). Anacetrapib, a New CETP Inhibitor: The New Tool for the Management of Dyslipidemias?. Diseases.

[111] Asztalos B.F., Collins D., Horvath K.V., Bloomfield H.E., Robins S.J., Schaefer E.J. (2008). Relation of gemfibrozil treatment and high-density lipoprotein subpopulation profile with cardiovascular events in the Veterans Affairs High-Density Lipoprotein Intervention Trial. Metabolism.

[112] Asztalos B.F. (2010). NLA Symposium on High Density Lipoproteins High-density lipoprotein particles, coronary heart disease, and niacin. J. Clin. Lipidol..

[113] Brown B.G., Zhao X.-Q., Chait A., Fisher L.D., Cheung M.C., Morse J.S., Dowdy A.A., Marino E.K., Bolson E.L., Alaupovic P. (2001). Simvastatin and Niacin, Antioxidant Vitamins, or the Combination for the Prevention of Coronary Disease. N. Engl. J. Med..

[114] Taylor A.J., Villines T.C., Stanek E.J., Devine P.J., Griffen L., Miller M., Weissman N.J., Turco M. (2009). Extended-Release Niacin or Ezetimibe and Carotid Intima–Media Thickness. N. Engl. J. Med..

[115] Julius U., Fischer S. (2013). Nicotinic acid as a lipid-modifying drug—A review. Atheroscler. Suppl..

[116] Bala I.M., Lifshits V.M., Sidel’nikova V.I. (1988). Granulocytic chalone and antichalone as homeostatic factors in the functional system of the blood in aseptic inflammation. Patol. Fiziol. Eksp. Ter..

[117] Carballo-Jane E., Chen Z., O’Neill E., Wang J., Burton C., Chang C.H., Chen X., Eveland S., Frantz-Wattley B., Gagen K. (2010). ApoA-I mimetic peptides promote pre-β HDL formation in vivo causing remodeling of HDL and triglyceride accumulation at higher dose. Bioorg. Med. Chem..

[118] Bełtowski J. (2008). Liver X Receptors (LXR) as Therapeutic Targets in Dyslipidemia. Cardiovasc. Ther..

[119] Mencarelli A., Fiorucci S. (2010). FXR an emerging therapeutic target for the treatment of atherosclerosis. J. Cell. Mol. Med..

[120] Rayner K.J., Moore K.J. (2014). MicroRNA Control of High-Density Lipoprotein Metabolism and Function. Circ. Res..

[121] Navab M., Anantharamaiah G.M., Reddy S.T., Hama S., Hough G., Grijalva V.R., Yu N., Ansell B.J., Datta G., Garber D.W. (2005). Apolipoprotein A-I Mimetic Peptides. Arterioscler. Thromb. Vasc. Biol..

[122] Remaley A.T., Amar M., Sviridov D. (2008). HDL-replacement therapy: Mechanism of action, types of agents and potential clinical indications. Expert Rev. Cardiovasc. Ther..

[123] Verschuren L., de Vries-van der Weij J., Zadelaar S., Kleemann R., Kooistra T. (2009). LXR agonist suppresses atherosclerotic lesion growth and promotes lesion regression in apoE*3Leiden mice: Time course and mechanisms. J. Lipid Res..

[124] Hafiane A., Genest J. (2013). HDL, Atherosclerosis, and Emerging Therapies. Cholesterol.

[125] Ouimet M., Ediriweera H., Afonso M.S., Ramkhelawon B., Singaravelu R., Liao X., Bandler R.C., Rahman K., Fisher E.A., Rayner K.J. (2017). microRNA-33 Regulates Macrophage Autophagy in Atherosclerosis. Arterioscler. Thromb. Vasc. Biol..

[126] Van de Woestijne A.P., van der Graaf Y., Liem A.-H., Cramer M.J.M., Westerink J., Visseren F.L.J., SMART Study Group (2013). Low High-Density Lipoprotein Cholesterol Is Not a Risk Factor for Recurrent Vascular Events in Patients with Vascular Disease on Intensive Lipid-Lowering Medication. J. Am. Coll. Cardiol..

[127] Pirillo A., Tibolla G., Norata G.D., Catapano A.L. (2014). HDL: To Treat or Not To Treat?. Curr. Atheroscler. Rep..

[128] Estrada-Luna D., Martínez-Hinojosa E., Cancino-Diaz J.C., Belefant-Miller H., López-Rodríguez G., Betanzos-Cabrera G. (2018). Daily supplementation with fresh pomegranate juice increases paraoxonase 1 expression and activity in mice fed a high-fat diet. Eur. J. Nutr..

[129] Stowe C.B. (2011). The effects of pomegranate juice consumption on blood pressure and cardiovascular health. Complement. Ther. Clin. Pract..

[130] Basu A., Penugonda K. (2009). Pomegranate juice: A heart-healthy fruit juice. Nutr. Rev..

[131] Aviram M., Rosenblat M. (2004). Paraoxonases 1, 2, and 3, oxidative stress, and macrophage foam cell formation during atherosclerosis development. Free Radic. Biol. Med..

[132] Arulselvan P., Fard M.T., Tan W.S., Gothai S., Fakurazi S., Norhaizan M.E., Kumar S.S. (2016). Role of Antioxidants and Natural Products in Inflammation. Oxid. Med. Cell. Longev..

[133] Mackness M.I., Arrol S., Durrington P.N. (1991). Paraoxonase prevents accumulation of lipoperoxides in low-density lipoprotein. FEBS Lett..

[134] Oda M.N., Bielicki J.K., Ho T.T., Berger T., Rubin E.M., Forte T.M. (2002). Paraoxonase 1 Overexpression in Mice and Its Effect on High-Density Lipoproteins. Biochem. Biophys. Res. Commun..

[135] Ikhlef S., Berrougui H., Kamtchueng Simo O., Zerif E., Khalil A. (2017). Human paraoxonase 1 overexpression in mice stimulates HDL cholesterol efflux and reverse cholesterol transport. PLoS ONE.

[136] Bhatia S., Shukla R., Venkata Madhu S., Kaur Gambhir J., Madhava Prabhu K. (2003). Antioxidant status, lipid peroxidation and nitric oxide end products in patients of type 2 diabetes mellitus with nephropathy. Clin. Biochem..

[137] Rosenblat M., Hayek T., Aviram M. (2006). Anti-oxidative effects of pomegranate juice (PJ) consumption by diabetic patients on serum and on macrophages. Atherosclerosis.

[138] Aviram M., Dornfeld L., Rosenblat M., Volkova N., Kaplan M., Coleman R., Hayek T., Presser D., Fuhrman B. (2000). Pomegranate juice consumption reduces oxidative stress, atherogenic modifications to LDL, and platelet aggregation: Studies in humans and in atherosclerotic apolipoprotein E–deficient mice. Am. J. Clin. Nutr..

[139] Mackness M., Mackness B. (2004). Paraoxonase 1 and atherosclerosis: Is the gene or the protein more important?. Free Radic. Biol. Med..

[140] Leckey L.C., Garige M., Varatharajalu R., Gong M., Nagata T., Spurney C.F., Lakshman R.M. (2010). Quercetin and Ethanol Attenuate the Progression of Atherosclerotic Plaques with Concomitant Up Regulation of Paraoxonase1 (PON1) Gene Expression and PON1 Activity in LDLR−/− Mice. Alcohol. Clin. Exp. Res..

[141] Kaur H.D., Bansal M.P. (2009). Studies on HDL associated enzymes under experimental hypercholesterolemia: Possible modulation on selenium supplementation. Lipids Health Dis..

[142] Koukos G., Chroni A., Duka A., Kardassis D., Zannis V.I. (2007). Naturally occurring and bioengineered apoA-I mutations that inhibit the conversion of discoidal to spherical HDL: The abnormal HDL phenotypes can be corrected by treatment with LCAT. Biochem. J..

[143] Fawole O.A., Opara U.L. (2016). Stability of total phenolic concentration and antioxidant capacity of extracts from pomegranate co-products subjected to in vitro digestion. BMC Complement. Altern. Med..

[144] Lampe J.W. (1999). Health effects of vegetables and fruit: Assessing mechanisms of action in human experimental studies. Am. J. Clin. Nutr..

[145] Aviram M., Volkova N., Coleman R., Dreher M., Reddy M.K., Ferreira D., Rosenblat M. (2008). Pomegranate Phenolics from the Peels, Arils, and Flowers Are Antiatherogenic: Studies in Vivo in Atherosclerotic Apolipoprotein E-Deficient (E0) Mice and in Vitro in Cultured Macrophages and Lipoproteins. J. Agric. Food Chem..

[146] Vilahur G., Padró T., Casaní L., Mendieta G., López J.A., Streitenberger S., Badimon L. (2015). Polyphenol-enriched Diet Prevents Coronary Endothelial Dysfunction by Activating the Akt/eNOS Pathway. Rev. Esp. Cardiol. Engl. Ed..

[147] Badimon L., Martínez-González J., Llorente-Cortés V., Rodríguez C., Padró T. (2006). Cell biology and lipoproteins in atherosclerosis. Curr. Mol. Med..

[148] Sarwar N., Sattar N. (2009). Triglycerides and coronary heart disease: Have recent insights yielded conclusive answers?. Curr. Opin. Lipidol..

[149] Kjaergaard A.G., Dige A., Krog J., Tønnesen E., Wogensen L. (2013). Soluble adhesion molecules correlate with surface expression in an in vitro model of endothelial activation. Basic Clin. Pharmacol. Toxicol..

[150] Perségol L., Darabi M., Dauteuille C., Lhomme M., Chantepie S., Rye K.-A., Therond P., Chapman M.J., Salvayre R., Nègre-Salvayre A. (2018). Small dense HDLs display potent vasorelaxing activity, reflecting their elevated content of sphingosine-1-phosphate. J. Lipid Res..

[151] Qi J., Zheng J.-B., Ai W.-T., Yao X.-W., Liang L., Cheng G., Shou X.-L., Sun C.-F. (2017). Felodipine inhibits ox-LDL-induced reactive oxygen species production and inflammation in human umbilical vein endothelial cells. Mol. Med. Rep..

[152] Lloyd-Jones D.M., Larson M.G., Beiser A., Levy D. (1999). Lifetime risk of developing coronary heart disease. Lancet.

[153] Stalenhoef A.F., de Graaf J. (2008). Association of fasting and nonfasting serum triglycerides with cardiovascular disease and the role of remnant-like lipoproteins and small dense LDL. Curr. Opin. Lipidol..

[154] Jagla A., Schrezenmeir J. (2001). Postprandial triglycerides and endothelial function. Exp. Clin. Endocrinol. Diabetes Off. J. Ger. Soc. Endocrinol. Ger. Diabetes Assoc..

[155] Van Eck M., Bos I.S.T., Kaminski W.E., Orso E., Rothe G., Twisk J., Bottcher A., Van Amersfoort E.S., Christiansen-Weber T.A., Fung-Leung W.-P. (2002). Leukocyte ABCA1 controls susceptibility to atherosclerosis and macrophage recruitment into tissues. Proc. Natl. Acad. Sci. USA.

[156] Kuai R., Li D., Chen Y.E., Moon J.J., Schwendeman A. (2016). High-Density Lipoproteins: Nature’s Multifunctional Nanoparticles. ACS Nano.

[157] Chung R.W.S., Leanderson P., Lundberg A.K., Jonasson L. (2017). Lutein exerts anti-inflammatory effects in patients with coronary artery disease. Atherosclerosis.

[158] Shen C.-Y., Jiang J.-G., Huang C.-L., Zhu W., Zheng C.-Y. (2017). Polyphenols from Blossoms of *Citrus aurantium* L. var. amara Engl. Show Significant Anti-Complement and Anti-Inflammatory Effects. J. Agric. Food Chem..

[159] Danesi F., Ferguson L. (2017). Could Pomegranate Juice Help in the Control of Inflammatory Diseases?. Nutrients.

[160] Bhatt D.L. (2008). Anti-Inflammatory Agents and Antioxidants as a Possible “Third Great Wave” in Cardiovascular Secondary Prevention. Am. J. Cardiol..

[161] Siasos G., Tousoulis D., Tsigkou V., Kokkou E., Oikonomou E., Vavuranakis M., Basdra E.K., Papavassiliou A.G., Stefanadis C. (2013). Flavonoids in atherosclerosis: An overview of their mechanisms of action. Curr. Med. Chem..

[162] Dauchet L., Amouyel P., Dallongeville J. (2009). Fruits, vegetables and coronary heart disease. Nat. Rev. Cardiol..

[163] Arós F., Estruch R. (2013). Mediterranean Diet and Cardiovascular Prevention. Rev. Esp. Cardiol. Engl. Ed..

[164] Aviram M., Rosenblat M., Gaitini D., Nitecki S., Hoffman A., Dornfeld L., Volkova N., Presser D., Attias J., Liker H. (2004). Pomegranate juice consumption for 3 years by patients with carotid artery stenosis reduces common carotid intima-media thickness, blood pressure and LDL oxidation. Clin. Nutr..

[165] Delgado N.T.B., Rouver W.N., Freitas-Lima L.C., de Paula T.D.-C., Duarte A., Silva J.F., Lemos V.S., Santos A.M.C., Mauad H., Santos R.L. (2016). Pomegranate Extract Enhances Endothelium-Dependent Coronary Relaxation in Isolated Perfused Hearts from Spontaneously Hypertensive Ovariectomized Rats. Front. Pharmacol..

[166] Olivero-David R., Ruiz-Roso M.B., Caporaso N., Perez-Olleros L., De Las Heras N., Lahera V., Ruiz-Roso B. (2018). In vivo bioavailability of polyphenols from grape by-product extracts, and effect on lipemia of normocholesterolemic Wistar rats. J. Sci. Food Agric..

[167] Nauman M., Kale R.K., Singh R.P. (2018). Polyphenols of Salix aegyptiaca modulate the activities of drug metabolizing and antioxidant enzymes, and level of lipid peroxidation. BMC Complement. Altern. Med..

[168] Wang L., Zeng B., Liu Z., Liao Z., Zhong Q., Gu L., Wei H., Fang X. (2018). Green Tea Polyphenols Modulate Colonic Microbiota Diversity and Lipid Metabolism in High-Fat Diet Treated HFA Mice. J. Food Sci..

[169] Kuvin J.T., Dave D.M., Sliney K.A., Mooney P., Patel A.R., Kimmelstiel C.D., Karas R.H. (2006). Effects of extended-release niacin on lipoprotein particle size, distribution, and inflammatory markers in patients with coronary artery disease. Am. J. Cardiol..

[170] van der Hoorn J.W.A., de Haan W., Berbee J.F.P., Havekes L.M., Jukema J.W., Rensen P.C.N., Princen H.M.G. (2008). Niacin Increases HDL by Reducing Hepatic Expression and Plasma Levels of Cholesteryl Ester Transfer Protein in APOE*3Leiden.CETP Mice. Arterioscler. Thromb. Vasc. Biol..

[171] Vilahur G., Badimon L. (2013). Antiplatelet properties of natural products. Vasc. Pharmacol..

[172] Chang C.L., Deckelbaum R.J. (2013). Omega-3 fatty acids: Mechanisms underlying “protective effects” in atherosclerosis. Curr. Opin. Lipidol..

[173] Catalán U., Fernández-Castillejo S., Pons L., Heras M., Aragonés G., Anglès N., Morelló J.-R., Solà R. (2012). Alpha-tocopherol and BAY 11-7082 reduce vascular cell adhesion molecule in human aortic endothelial cells. J. Vasc. Res..

[174] van Dam B., van Hinsbergh V.W.M., Stehouwer C.D.A., Versteilen A., Dekker H., Buytenhek R., Princen H.M., Schalkwijk C.G. (2003). Vitamin E inhibits lipid peroxidation-induced adhesion molecule expression in endothelial cells and decreases soluble cell adhesion molecules in healthy subjects. Cardiovasc. Res..

[175] Palozza P., Parrone N., Simone R.E., Catalano A. (2010). Lycopene in atherosclerosis prevention: An integrated scheme of the potential mechanisms of action from cell culture studies. Arch. Biochem. Biophys..

[176] Shah T.A., Parikh M., Patel K.V., Patel K.G., Joshi C.G., Gandhi T.R. (2016). Evaluation of the effect of Punica granatum juice and punicalagin on NFκB modulation in inflammatory bowel disease. Mol. Cell. Biochem..

[177] Aviram M., Dornfeld L., Kaplan M., Coleman R., Gaitini D., Nitecki S., Hofman A., Rosenblat M., Volkova N., Presser D. (2002). Pomegranate juice flavonoids inhibit low-density lipoprotein oxidation and cardiovascular diseases: Studies in atherosclerotic mice and in humans. Drugs Exp. Clin. Res..

